# Cerium Oxide on a Fluorinated Carbon-Based Electrode as a Promising Catalyst for Hypochlorite Production

**DOI:** 10.1021/acsomega.2c04248

**Published:** 2022-10-10

**Authors:** María Isabel Alvarado-Ávila, Esteban Toledo-Carrillo, Joydeep Dutta

**Affiliations:** Functional NanoMaterials Group, Department of Applied Physics, School of Engineering Sciences, KTH Royal Institute of Technology, Hannes Alfvéns väg 12, 11419Stockholm, Sweden

## Abstract

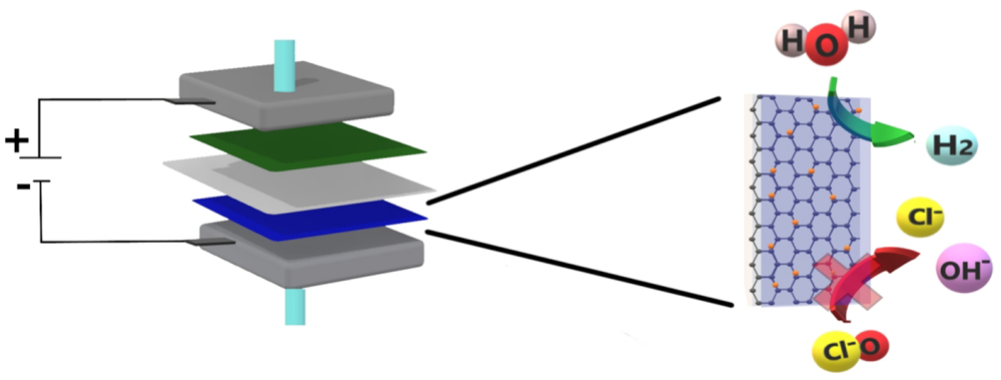

Sodium hypochlorite (NaOCl) is widely used as a disinfectant agent for water treatment and surface cleaning. A straightforward way to produce NaOCl is by the electrolysis of an aqueous sodium chloride (NaCl) solution. This process presents several side reactions decreasing its efficiency with hypochlorite reduction on the cathode surface being one of the main detrimental reactions. In this work, we have studied carbon-based electrodes modified with cerium oxide (CeO_2_), fluorine, and platinum nanoparticles as cathodes for hypochlorite production. Fluorination was carried out electrochemically; the polyol method was used to synthesize platinum nanoparticles; and the hydrothermal process was applied to form a CeO_2_ layer. Scanning electron microscopy, FTIR, and inductively coupled plasma (ICP) indicated the presence of cerium oxide as a film, fluorine groups on the substrate, and a load of 3.2 mg/cm^2^ of platinum nanoparticles and 2.7 mg/cm^2^ of CeO_2_. From electrochemical impedance spectroscopy, it was possible to demonstrate that incorporating platinum and fluorine decreases the charge transfer resistance by 16% and 28%, respectively. Linear sweep voltammetry showed a significant decrease in hypochlorite reduction when the substrate was doped with fluorine from −16.6 mA/cm^2^ at −0.6 V to −9.64 mA/cm^2^ that further reduced to −8.78 mA/cm^2^ with cerium oxide covered fluorinated electrodes. The performance of the cathode materials during hypochlorite production improved by 80% compared with pristine activated carbon cloth (ACC) electrodes. The improvement toward hindering NaOCl reduction is probably caused by the incorporation of a partial negative charge upon doping with fluorine.

## 1 Introduction

Aqueous sodium hypochlorite (NaOCl) exists as hypochlorite ion (OCl^–^), also known as free chlorine, a strong oxidizing agent widely used in water treatment, as a disinfectant in households, hospitals, and the food industry. NaOCl has also been applied to prevent membrane fouling in ultrafiltration membrane separation processes^1^ and surface disinfection from several types of viruses and bacteria like *Staphylococcus aureus*,^2^*Escherichia coli*,^3,4^*Bacillus subtilis*,^5,6^ and so on. Hypochlorite is a prominent disinfectant used in hospitals, not only to clean surfaces like metals, glass, and plastics where viruses and bacteria can persist for up 9 days^7^ but also to treat hospital waste and wastewater^8^ to avoid the spread of viruses and infections.

Sodium hypochlorite can be produced by two methods: chemically and electrochemically. Direct reaction between chlorine gas (Cl_2_) and caustic soda (NaOH), reaction 1, leads to hypochlorite formation. During the sodium chloride (NaCl) electrolysis, hydrogen gas (H_2_) is produced on the cathode, as shown in reaction 2, and chlorine gas (Cl_2_) is evolved on the anode, as shown in reaction 3. Chlorine dissolves in the bulk solution and hydrolyzes to form hypochlorous acid (HOCl), followed by the formation of hypochlorite (OCl^–^), as shown in reactions 4 and 5.
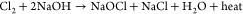
1
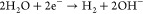
2
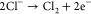
3
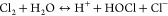
4
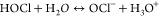
5
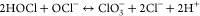
6
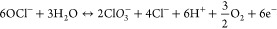
7
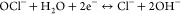
8

During the electrolysis process, there are some losses^9^ due to side reactions involving hypochlorite species. The first side reactions occur due to the formation of chlorates in the bulk solution (reaction 6) or due to hypochlorite oxidation on the anode (reaction 7). This can be avoided by adjusting the pH to above 6. The second reaction related to hypochlorite reduction at the cathode (reaction 8) is more complex to manage. Generally hexavalent chromium (Cr(VI)) salts are added during chlorate production to hinder hypochlorite reduction through the formation of a film of chromium oxide/hydroxide Cr(OH)_3_ that makes the cathode more selective to hydrogen evolution.^10,11^ Cr(VI) salts also act as a buffer by keeping the pH between 6 and 6.5 and protecting the cathode from corrosion.^12^ However, the toxicity of Cr(VI) toward human health, especially the respiratory tract, liver, immune system, and so on,^13^ has limited its use in the last years, motivating research for other alternative solutions.

Commercially, sodium chloride electrolysis for chlorate and hypochlorite production uses steel cathodes, platinum or titanium electrodes showing a decent efficiency for hydrogen evolution, but the hypochlorite reduction reaction is still a problem.^14^ Other solutions reported in the literature to reduce hypochlorite reduction include using alternative salts like yttrium chloride,^15^ sodium permanganate,^16^ and sodium vanadate.^17^ Even though this inhibits the hypochlorite reduction due to oxide/hydroxide film formation, some limitations like the solubility of yttrium chloride compromising the film deposition and the addition of sodium vanadate that doubles the hypochlorite to oxygen decomposition in the bulk solution restrict their widespread application. Manganese oxide film deposited on the cathode due to the addition of permanganate often leads to a continuous growth that peel out from the electrode during the process. Thus, researchers are focusing more on developing cathode materials with selective properties for hydrogen production and reducing the rate of side reactions like hypochlorite reduction. The ex situ electrodeposition of chromium oxide/hydroxide film has been considered an alternative to avoid the incorporation of Cr(VI) in the bulk solution for reducing ClO^–^ reduction.^18^ Lačnjevac et al. studied the deposition of chromium–molybdenum oxide film coated titanium electrodes that showed a decrease in the hypochlorite reduction rate and increasing selectivity of the cathode for the hydrogen evolution reaction.^19^ However, the manufacturing process for these films involves using hexavalent chromium salts that are toxic.

Cerium oxide has been proposed as an alternative for controlling hypochlorite reduction in recent times due to its selectivity property, finding application in electrodes of fuel cells^20,21^ and hydrogen peroxide production.^22^ Endrődi et al. studied in situ and ex situ deposition of cerium oxide film on platinum cathodes for chlorate production, finding that ex situ formation leads to an effective cathode performance by decreasing the rate of hypochlorite reduction; in situ formation was found to lead to the precipitation of cerium hydroxide accelerating hypochlorite decomposition. Cerium is known to have excellent mechanical stability and corrosion resistance under alkaline conditions^23,24^ making it suitable for hypochlorite production where pH is kept between 8 and 9.

Titanium and platinum electrodes are generally used as materials for cathode fabrication, but their high-cost limits larger-scale application. As an alternative, porous carbon materials like activated carbon are gaining attention to be used as a substrate for oxygen reduction reaction^25,26^ and alcohol oxidations^27,28^ due to their economic feasibility, chemical inertness to most electrolytes, and the possibility of obtaining a high surface area as well as a wide operating potential range. However, problems due to carbon oxidation are still an issue.^29^ Incorporating heteroatoms like fluorine and nitrogen has been shown to reduce carbon oxidation improving its stability during electrolysis processes.^30^ In addition, it has been found that fluorine increases the active sites on carbon electrodes due to its high electronegativity and the polarization of adjacent carbon leading to enhanced catalytic activity of the electrodes.^31,32^ Platinum nanoparticles are well-known to increase the hydrogen evolution activity and the conductivity of the supporting material, so they are widely used in electrolysis processes. Considering the above, we propose that modified carbon-based electrodes can fulfill the requirements for chlorate and hypochlorite production in undivided cells, decreasing the process cost and avoiding the use of Cr(VI) components to eliminate hypochlorite reduction. In the present study, nanocomposite coatings on fluorinated activated carbon with cerium oxide have been applied, characterized, and tested to avoid hypochlorite reduction and finally used as cathodes for hypochlorite generation.

## 2 Experimental Section

### 2.1 Chemical Used

Sodium chloride salt (NaCl) was purchased from VWR International (Leuven, Belgium), >99.5 pure sodium, sodium hydroxide (NaOH), cerium(II) chloride (CeCl_2_), and ammonia solution 25% were purchased from Merck (Darmstadt, Germany). Citric acid was obtained from J. T. Baker, Baker Analyzed Reagent (Deventer, Holland), 50% hydrofluoric acid (HF) was from BASF Chemicals Company (Ludwigshafen, Germany), 50% ethylene glycol ((CH_2_OH)_2_) was obtained from DGR industrial products (Livermore, United States of America). Chloroplatinic acid (H_2_PtCl_6_) was purchased from Sigma-Aldrich (Hamburg, Germany). All solutions used in the experiments were prepared with deionized water.

### 2.2 Fabrication of the Electrodes

As received single-weaved activated carbon cloth (ACC), Zorflex FM10 (surface density 220 g/m^2^, thickness 0.6 mm, surface area ∼1100 m^2^/g), purchased from Chemviron Carbon Ltd. (Houghton-le-Spring, U.K.), was cleaned with 3 M HNO_3_ at 80 °C for 3 h and then thoroughly washed with deionized water until neutral pH, dried overnight in an oven kept at 60 °C, and then stored in a desiccator until further use. 210B industrial-grade graphite foil was obtained from Minseal Corporation (thickness 0.18 mm) and was used after cleaning thoroughly with isopropanol.

Four types of electrodes were prepared: pristine activated carbon cloth (ACC), fluorinated activated carbon cloth coated with cerium oxide (CeO_2_/ACC-F), activated carbon covered with cerium oxide (CeO_2_/ACC), and activated carbon cloth with platinum nanoparticles dispersed on cerium oxide as a coating layer (CeO_2_/ACC-Pt).

Electrochemical fluorination of ACC was carried out using an aqueous solution of hydrofluoric acid (20%). ACC was the working electrode, and a graphite foil was used as the counter electrode. An external potential of 10 V was applied for 15 min as described in detail elsewhere.^33^ Cerium oxide was prepared by a hydrothermal process following a method described in the literature with slight modifications.^34^ In brief, equimolar (1 M) volumes of cerium chloride (CeCl_2_) and citric acid (C_6_H_8_O_7_) were mixed in an aqueous solution, where CeCl_2_ acts as the oxide precursor, and citric acid plays the role of a capping agent stabilizing the particles during its germination and growth. 20 μL/cm^2^ of this precursor was drop casted on clean ACC substrates and dried in atmospheric conditions at 60 °C. The samples were immersed under continuous stirring in an excess amount of 3.0 M ammonia solution at 50 °C for 24 h. An ammonia solution acts as a source of hydroxyl ions, favoring the Ce^3+^/Ce^4+^ oxidation. The excess ammonia keeps the pH constant during the precipitation process to obtain uniform particles.^35^ Following this step, the solution and the ACC were transferred to a Teflon-lined autoclave and keep it at 80 °C for 24 h. Once the process was completed, the ACC samples were washed several times with deionized water and dried at 60 °C overnight. The fluorinated activated carbon cloth covered with cerium oxide were thus obtained.

Platinum (Pt) nanoparticles were obtained by a polyol method, by adding a solution of 600 ppm of hexachloroplatinic acid (H_2_PtCl_6_) in ethanol to the substrate followed by drying at 60 °C for 10 min, and then it was immersed in an ethylene glycol bath at 160 °C for 10 min. After the electrode was washed with abundant deionized water, it was dried again at 60 °C for 24 h. A cerium oxide film to cover the platinum nanoparticles was also obtained using the above-described method.

### 2.3 Electrode Surface Characterization

The microstructure and the elemental chemical analysis of the electrode surfaces were studied using a ZEISS Ultra-55 scanning electron microscope (SEM) equipped with energy-dispersive X-ray spectroscopy (SEM-EDX), with a working voltage of 10 kV (GEMINI Ultra 55, Carl Zeiss, Oberkochen, Germany). Fourier Transform Infrared spectroscopy (FTIR) was used to distinguish the functional groups on the modified activated carbon cloth substrate with Nicolet iS10 FTIR spectrometer in the attenuated total reflectance (ATR) mode (Thermo Fisher Scientific, Waltham, MA). Thermo Scientific iCAP 6500 induced coupled plasma-optical emission spectroscopy (ICP-OES) was used to determine the electrode platinum content by measuring the platinum difference in the precursor solution before and after the polyol process.

X-ray diffraction analysis (XRD) was used to determine the crystal structure of the cerium oxide layer synthesized on activated carbon cloth. The XRD patterns were collected by an X-ray diffractometer (X’pert PRO, PANalytical B.V., Netherlands) with Cu Kα (λ = 1.5406 Å) monochromatic radiation, scanning in the 2θ range from 10° to 80°. The crystallite size was determined using the Scherrer equation (eq 9), where “*d*” is the crystallite size, “λ” is the X-ray wavelength, “β” is the full width at half the maximum intensity of the peak, and “θ” is the Bragg angle or the peak position.
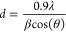
9

### 2.4 Electrochemical Characterization

The electrochemical tests were performed in a typical three-electrode cell with Interface 1010E potentiostat (Gamry Instruments, Warminster, PA) at room temperature. The electrolyte used was 2 M NaCl + 80 mM NaClO at pH 11, with platinum wire as the counter electrode. Linear sweep voltammetry (LSV) was studied from 0.4 to −2.0 V at a scan rate of 2 mV/s. Cyclic voltammetry (CV) was run between 0.2 and −0.2 V; five potential cycles were performed at scan rates of 5, 10, 20, and 50 mV/s. Specific capacitance was calculated using eq 10, where “*C*” is the specific capacitance (F/g), “*m*” is the electrode mass (g), “*A*” correspond to the integrated area in the CV curve, “*k*” is the scan rate and “Δ*V*” the potential window.
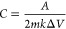
10

Double layer capacitance (*C*_DL_) was calculated from the CV curves measured at 5, 10, and 20 mV/s scan rate, plotting the charging current at −0.05 V (where no reactions are expected to take place) with respect to the scan rate, that yields a straight line with the slope showing the double layer capacitance.

Electrochemical impedance spectra (EIS) were collected with open circuit potential after stabilization of the system. A potential perturbation of 10 mV_rms_ was applied to acquire the impedance spectra in the frequency range of 100 kHz to 10 mHz. All the potentials were measured with Ag/AgCl (saturated KCl) reference electrode.

### 2.5 Hypochlorite Production

For hypochlorite production, a flat electrolytic cell was used where graphite sheets acted as the current collectors. The cell was built using 10 mm thick transparent poly(methyl methacrylate) (PMMA) sheets with point-to-point architecture comprising an inlet/outlet point centered at the top of the unit connected to four other circular inlet/outlet points located at the four corners of the square sheet, similar to a capacitive deionization (CDI) cell described elsewhere.^36^ A cellulose paper sheet (∼200 μm thickness) separated the electrodes of 5 × 5 cm^2^ dimensions, and a current density of 10 mA/cm^2^ was provided through PeakTech DC dual power supply 6210. The experiment was executed in a batch mode using 1 L of a 30 g/L sodium chloride solution, and the working voltage was continuously recorded by a KEITHLEY 2110 digital multimeter (Cleveland, OH). The pH was controlled and maintained over 9. Samples were extracted at different time intervals and diluted 25 times. Salt solutions with lower concentrations (10 g/L and 20 g/L) were also tested with the optimized CeO_2_/ACC-F electrode for hypochlorite generation.

### 2.6 Free Chlorine Concentration Measurement

Hypochlorite ion or free chlorine concentration was measured with a PerkinElmer Lambda 750 UV/vis spectrophotometer, using a test kit as per the standard ISO 7393-2:2018 protocol. A calibration curve was determined (as presented in Figure S1), and the standard solutions were prepared from a hypochlorite solution (13% concentration) by Iodometric titration.

## 3 Results and Discussion

**Figure 1:**
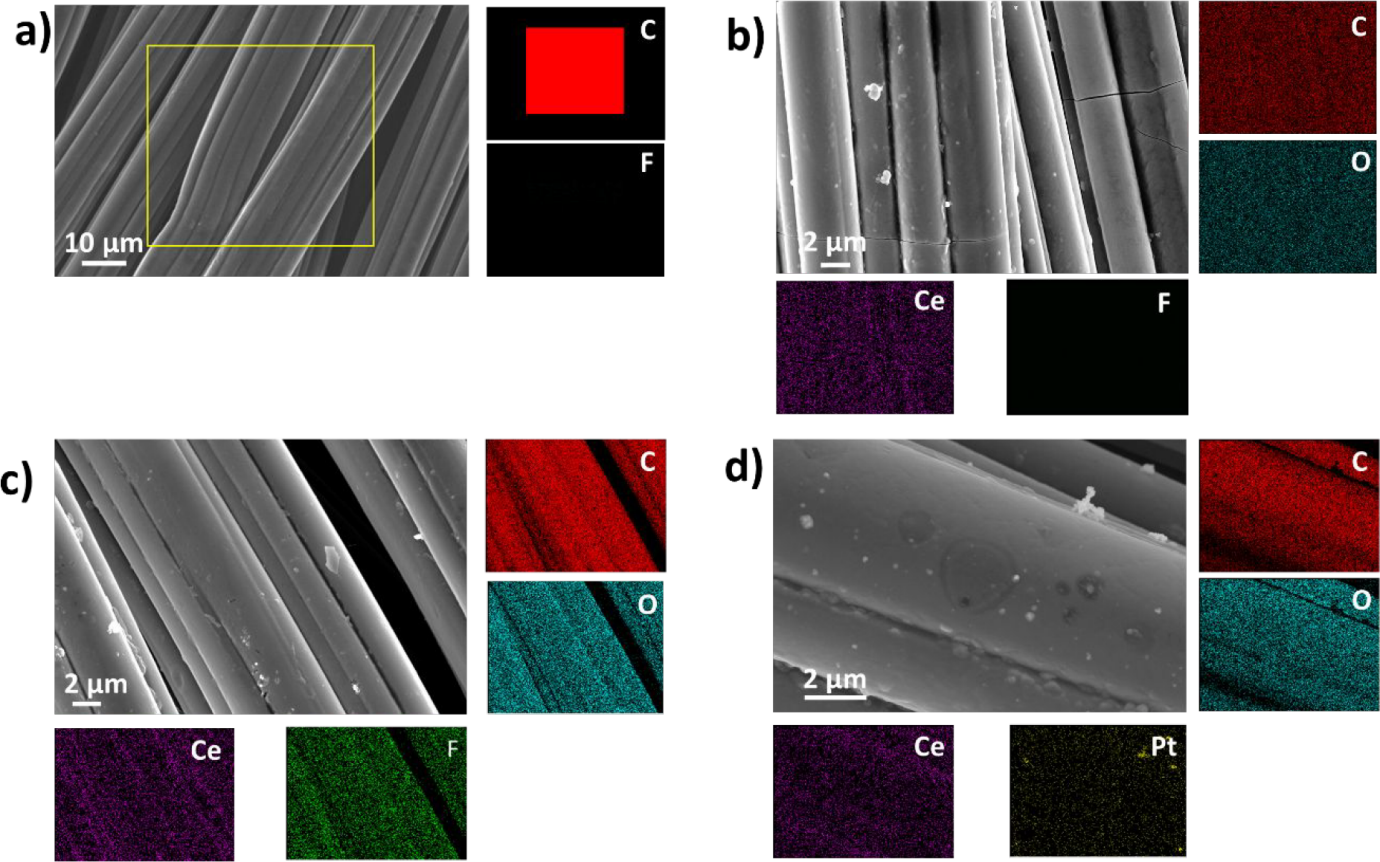
Scanning electron micrographs of the electrodes (a) pristine activated carbon cloth (ACC), (b) cerium oxide on activated carbon cloth (CeO_2_/ACC), (c) cerium oxide on fluorinated activated carbon cloth (CeO_2_/ACC-F), and (d) cerium oxide on activated carbon cloth with platinum nanoparticles dispersed on it (CeO_2_/ACC-Pt). The working voltage used corresponds to 10 kV.

Microstructures of the synthesized materials were examined by scanning electron microscopy, as shown in Figure 1. The corresponding EDX map illustrating the elemental distribution of cerium, oxygen, fluorine, and platinum can be found under each micrograph. Figure 1a corresponds to pristine ACC where EDS mapping corroborates the absence of any fluorine on the substrate. From Figure 1b–d, it is possible to notice that cerium oxide is homogeneously distributed on the activated carbon cloth fibers forming a thin layer covering the electrode. According to Masui et al., citric acid is adsorbed on the particles’ surfaces and limits further growth once the particles precipitate.^34^ From Figure S2, it is possible to notice that 15 min in ammonia solution was enough to form a layer of nanoparticles on the activated carbon. This layer becomes thicker after 1, 3, 6, 12, and 24 h of reaction time. A slight decrease in pH from 12.0 to 11.1 was found, which can explain the observation of agglomerates of 100 to 200 nm sizes formed due to perturbation of the colloid. Fluorine and platinum on the activated carbon cloth were also found in the EDS mapping, demonstrating successful fluorination of the activated carbon cloth and platinum loading. As observed in Figure 1c, fluorine is uniformly distributed together with carbon, cerium, and oxygen. Although the ACC was covered homogeneously, small cracks were found in the coatings. The cracks are uniaxial, on the longer side of the fibers between the fiber junctions. The appearance of crack is typical in the fabrication of thin films on inhomogeneous substrates because they are subjected to high residual stresses during thermal treatment, which cannot be dissipated entirely due to the differences between the elastic modulus of the deposited films and the substrate.^37^

Spherical platinum nanoparticles were deposited on CeO_2_/ACC-Pt electrode; the amount of platinum loaded on the ACC electrode before the growth of the CeO_2_ layer corresponded to 3.2 mg/cm^2^ (as determined from the results obtained from the precursor solutions before and after the process in the measurements carried out with ICP-OES). Some agglomerations of platinum nanoparticles were found, as shown in Figure S3a, and as can also be seen in the histogram presented in Figure S3b; the size of Pt nanoparticle’s are mainly between 10 to 40 nm (average Pt nanoparticle size ∼31 nm) agglomerated in 120–200 nm clusters. Some patches of uneven cerium oxide coatings were also found, especially in the electrode with platinum nanoparticles precipitated on ACC (Figure S4). According to Golunski et al., when the cerium oxide layer is formed on the Pt nanoparticles, a strong interaction between the platinum and cerium can produce CeO_2_ aggregates leading to a disruption in the layer distribution.^38^ Furthermore, hydrothermal synthesis may produce favorable conditions to increase the interaction between Pt and CeO_2_, which are beneficial to create additional active sites known to contribute to the redox reaction.^39^

**Figure 2:**
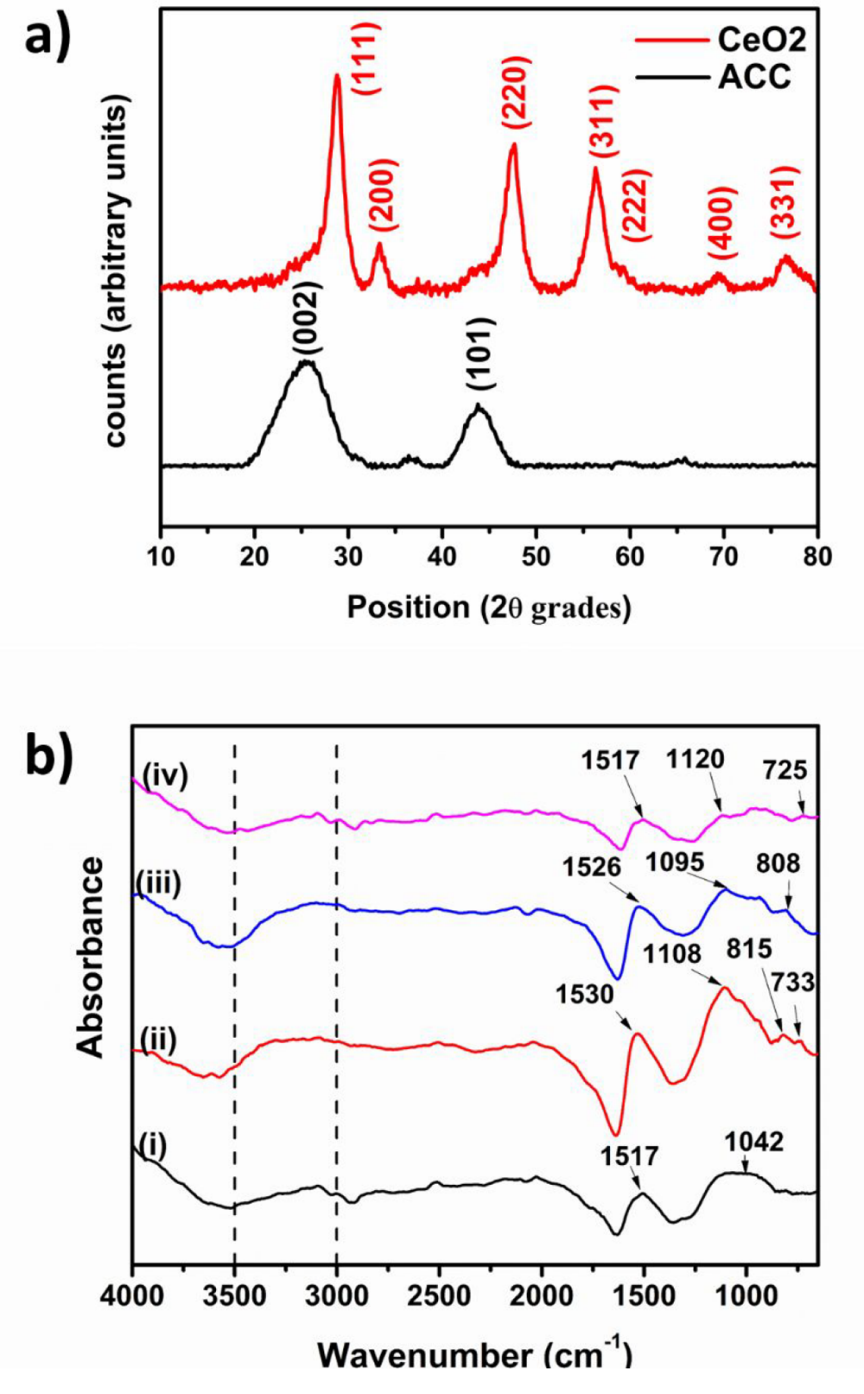
(a) X-ray diffraction pattern of activated carbon (ACC) and cerium oxide (CeO_2_) loaded on activated carbon cloth by the hydrothermal method. (b) FTIR spectra from (i) pretreated activated carbon cloth (ACC), (ii) cerium oxide deposited on ACC (CeO_2_/ACC), (iii) cerium oxide deposited on fluorinated ACC (CeO_2_/ACC-F), and (iv) cerium oxide on activated carbon cloth with platinum nanoparticles dispersed (CeO_2_/ACC-Pt).

Cerium oxide films were prepared by the hydrothermal method, and the mass loaded on the ACC electrode was determined to be 2.711 mg/cm^2^, as stimated from the mass difference of the substrate before and after treatment. Figure 2a shows the XRD pattern for activated carbon and cerium oxide coating ACC. Broad peaks at 26° and 43° can be ascribed to (002) and (101) planes of activated carbon cloth.^40^ The strong and sharp peaks found for the cerium oxide electrode matched the standard card JCPDS card no. 34-0394 for the ceria compound (CeO_2_) (Figure S5). No diffraction peaks other than ceria and ACC were registered, implying that highly pure CeO_2_ on ACC were synthesized. CeO_2_ has a crystal structure belonging to the *Fm*3*m* space group (fluorite-type cubic phase ceria structure) and a cell parameter of 5.4113 Å. Crystallite size was calculated from Scherrer equation (eq 9), showing an average size of 11.8 nm. Similar crystallite sizes are obtained during hydrothermal method synthesis (Table 1), where the chelating agent plays the major role during germination to control crystal growth. Citric acid provides citrate ions that can play multiple roles in the precipitation of nanoparticles, including as a reducing agent, a stabilizer, and a complexing agent. Since citric acid is a good chelating agent, normally smaller crystallite sizes are obtained, and due to the efficient stabilization of the particles during growth, uniform dispersion of particles is achieved in comparison to other chelating agents like ethylenediaminetetraacetate acid (EDTA) and ascorbic acid.^41^Table 1 also shows other synthesis methods reported in the literature. From here, it is possible to notice that the crystallite size is widely distributed between 3 nm and more than 40 nm depending on the conditions used. The synthesis temperature determines the crystallite size following the crystallization theory, wherein it is well-known that lower synthesis temperatures and low precursor concentrations leads to a small crystallite size.

**Table 1:** Comparison Table of Cerium Oxide Nanoparticles Synthesis Methods Found in the Literature

method	precursor/chelating agent	media	synthesis temperature (°C)	calcination (°C)	crystallite size (nm)	ref
hydrothermal	CeCl_2_/citric acid	3 M ammonia solution	80	-	11.8	this work
hydrothermal	Ce(NO_3_)_3_/ascorbic acid	ethanol/water	160	500	14	(42)
hydrothermal	Ce(NO_3_)_3_/citric acid	ethanol/water	160	500	11	(42)
hydrothermal	Ce(NO_3_)_3_/EDTA	ethanol/water	160	500	20.07	(42)
hydrothermal	Ce(NH_4_)_2_(NO_3_)_6_	0.4 ammonia solution	150	500	15.5	(43)
hydrothermal	Ce(NO_3_)_3_	ammonia	130	500	17.3	(44)
hydrothermal	Ce(NO_3_)_3_	ammonia	180	300	26.8	(45)
precipitation	Ce(NO_3_)_3_	3 M ammonia solution; O_2_/N_2_ mixture	30; 50; 70; 90	-	from 7.4 to 16.4	(35)
precipitation	Ce(NO_3_)_3_	3 M NaOH	450	450	9.8	(46)
precipitation	Ce(NO_3_)_3_	ammonia bicarbonate	RT	300; 400; 500; 600; 700	from 3.3 to 46.3	(47)
sol–gel	Ce(NO_3_)_3_	ethylene glycol	RT	850	43.9	(48)
sol–gel	(NH_4_)2Ce(NO_3_)_6_	HNO_3_	60	400	3.5	(49)
sol–gel	Ce(NO_3_)_3_	ethylene glycol	RT	600	4.84	(50)
sol–gel	Ce(NO_3_)_3_/oxalic acid	ethylene glycol, diethylamine	120	1100	13.13	(51)

Fourier transformed infrared (FTIR) spectra are shown in Figure 2b. The complete band list can be found in Table S1 in the Supporting Information. According to Asanov et al., C–F vibrations are associated with four frequency positions around 1230, 1132, 1095, and 1045 cm^–1^,^52^ as shown in Figure 2b(iii). The band around 1095 cm^–1^ corresponding to C–F vibrations are associated with a semicovalent bond.^53^ Ce–O stretching vibrations are found around 800 cm^–1^,^54,55^ and thus, the bands between 725 and 815 cm^–1^ found in CeO_2_/ACC, CeO_2_/ACC-F, and CeO_2_/ACC-Pt spectra can be associated to arise due to the presence of CeO_2_.

**Figure 3:**
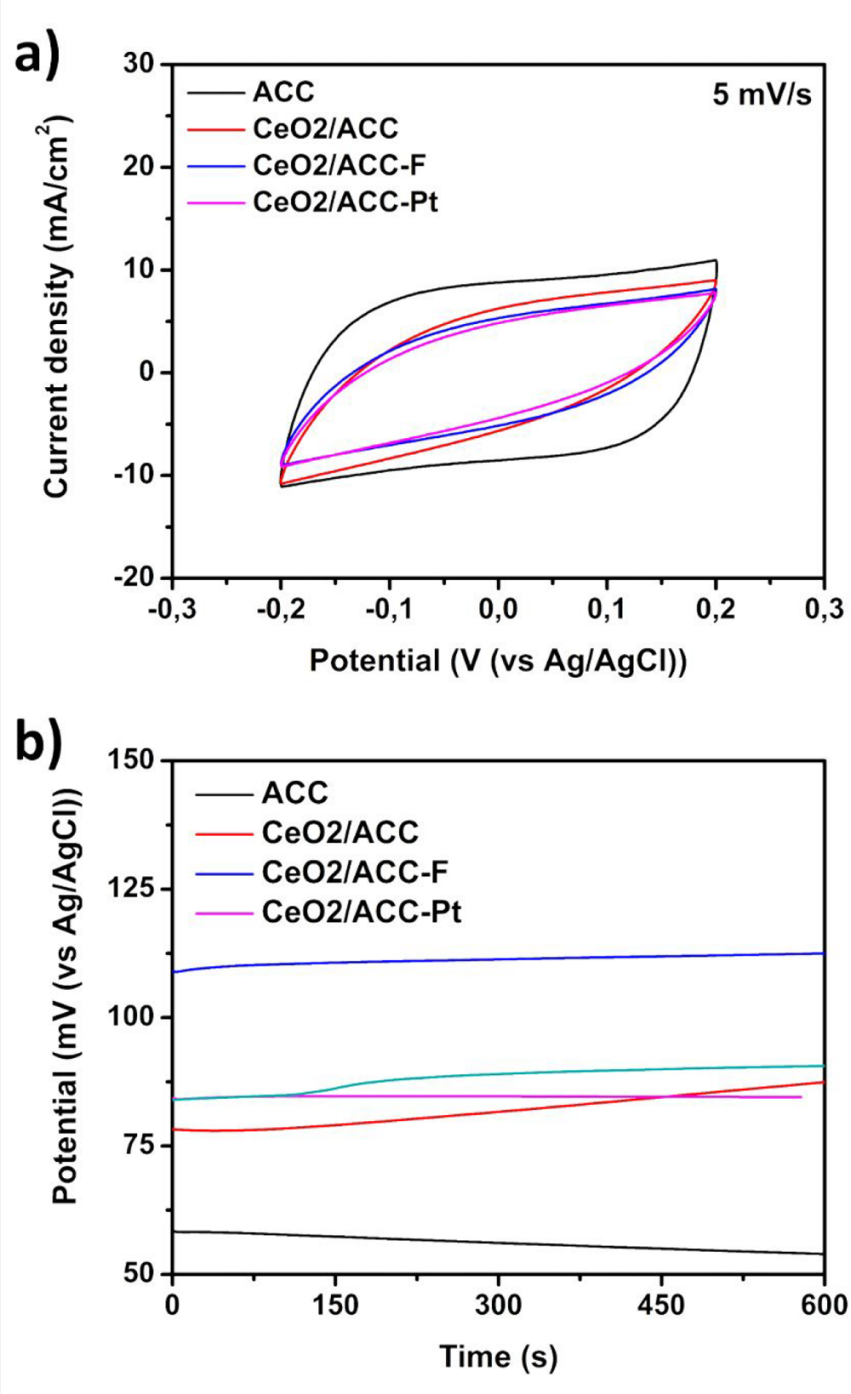
(a) Cyclic voltammetry curves for the different electrodes; Activated carbon (ACC), cerium oxide (CeO_2_) on ACC (CeO_2_/ACC), CeO_2_ on fluorinated ACC (CeO_2_/ACC-F), and CeO_2_ growth on platinum nanoparticles supported on ACC (CeO_2_/ACC-Pt), 5 mV/s scan rate. (b) Variation in the open circuit potential (OCP) over time for activated carbon cloth (ACC) and different electrodes modified with platinum, fluorine, and cerium oxide. The electrolyte used during the measurement corresponds to 2 M NaCl + 80 mM NaOCl, potentials measured with respect to the saturated Ag/AgCl reference electrode.

Cyclic voltammetry (CV) was carried out at 5, 10, 20, and 50 mV/s between −0.2 V and +0.2 V. CV curves at 5 mV/s are shown in Figure 3a (others are available in Figure S6 in the Supporting Information), presenting a typical shape of a double-layer capacitive behavior. Specific capacitance was calculated using eq 10, and the results are listed in Table 2. The specific capacitance at 5 mV/s can be summarized as ACC (65.99 F/g) > CeO_2_/ACC-F (37.42 F/g) > CeO_2_/ACC (34.86 F/g) > CeO_2_/ACC-Pt (29.24 F/g). When the scan rate is higher, these values tend to decrease due to the limitation in the interaction between the ions and the porous structure, but the trend is the same as indicated above. The presence of cerium oxide leads to the reduction of specific capacitance of ACC, but fluorination counters this to a certain degree that could be attributed to the incorporation of polar groups on the electrode surface.

**Table 2:** Specific Capacitance of Activated Carbon (ACC), Cerium Oxide Covered ACC (CeO_2_/ACC), Cerium Oxide on Fluorinated ACC (CeO_2_/ACC-F), and Cerium Oxide Coated Platinum Nanoparticles Supported on ACC (CeO_2_/ACC-Pt) Determined from Cyclic Voltammetry Curves at Different Scan Rates

	specific capacitance (F/g)			
electrode	5 mV/s	10 mV/s	20 mV/s	50 mV/s
ACC	65.998	47.988	23.994	8.014
CeO_2_/ACC	34.864	17.631	8.473	3.080
CeO_2_/ACC-F	37.422	20.638	11.01	4.661
CeO_2_/ACC-Pt	29.237	15.776	8.068	3.067

It is well-known that specific capacitance includes both the contribution of pseudocapacitance and double layer capacitance (C_DL_),^56^ where pseudocapacitance controls fast surface redox reactions while C_DL_ determines ion adsorption. Electrochemical surface area (ECSA) of the electrodes is thus proportional to C_DL_.^57,58^ So ECSA should follow the same trend as specific capacitance. Double layer capacitance was calculated from CV curves at a potential of −0.05 V and plotted against the scan rate, see Figure S7. Pristine ACC presented the highest double layer capacitance, as expected from specific capacitance values but is abruptly reduced for electrodes that were modified and coated with cerium oxide mainly due to the reduction in exposed surface. CeO_2_/ACC and CeO_2_/ACC-Pt electrode presented similar value of double layer capacitance, 56.18 and 60.70 mF/cm^2^, respectively. On the other hand, the C_DL_ of CeO_2_/ACC-F is 2 times higher than the other electrodes (127.43 mF/cm^2^).

Open circuit potential (OCP) measures the potential when no current passes through the electrode; OCP can provide information on the electrode’s surface charges. Figure 3b shows the OCP of the electrodes, an increase in the OCP value is associated with higher negative surface charges.^59^ Higher OCP was obtained for CeO_2_/ACC-F (approximately 112 mV), followed by ACC-F (90 mV), CeO_2_/ACC (87 mV), and CeO_2_/ACC-Pt (84 mV), and ACC (54 mV). Despite CeO_2_ being negatively charged at basic pH,^60^ adding platinum decreases the OCP only slightly, while fluorine dramatically increases the potential. Therefore, the addition of fluorine not only improved the conductivity of the electrodes due to the formation of semi-ionic or semicovalent C–F bonds^61^ but also the difference between carbon and fluorine electronegativity leads to the introduction of partial negatively charged surface as corroborated by the OCP measurements.

**Figure 4:**
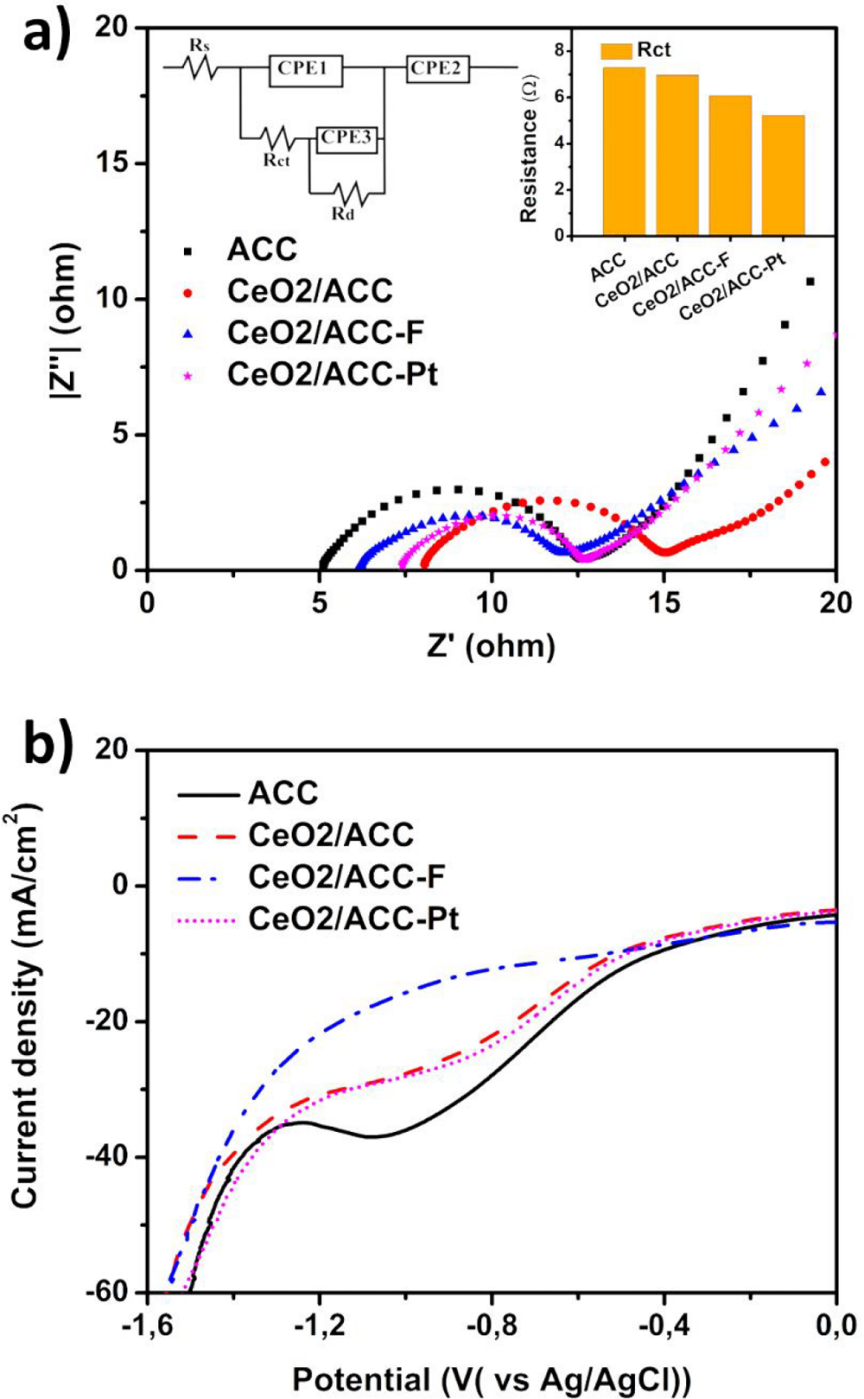
a) Nyquist plot for activated carbon cloth (ACC), Cerium oxide on ACC (CeO_2_/ACC), cerium oxide on fluorinated (CeO_2_/ACC-F), and cerium oxide loaded on platinum nanoparticles deposited on ACC (CeO_2_/ACC-Pt), Z′ and Z′′ are the real and imaginary impedance respectively. The bar plot in the inset corresponds to series resistance (*R*_s_) and charge transfer resistance (*R*_ct_) values obtained from fitted curves using the equivalent circuit shown in the left upside corner. (b) LSV in the cathodic direction at a scan rate of 2 mV/s. The electrolyte used during the measurement corresponded to 2 M NaCl + 80 mM NaClO. The potential was measured by using a saturated Ag/AgCl reference electrode.

Electrochemical kinetics were analyzed from electrochemical impedance spectroscopy (EIS). Figure 4a corresponds to the Nyquist plots of ACC, CeO_2_/ACC, CeO_2_/ACC-F, and CeO_2_/ACC-Pt electrodes. The shape of the Nyquist plots is associated with three main sections. First, a significant displacement of the plot in the real axis direction (*x*-axis), where the intersection with the *x*-axis at high frequencies corresponds to the series resistance (*R*_s_), which is more associated with the ionic conductivity of the electrolyte than with the electrode conductivity itself.^62,63^ Second, a semicircle appears at high frequencies related to a resistance-constant phase element (*R*_ct_-CPE) circuit where the CPE is associated with a nonideal capacitance usually attributed to rough or porous electrodes like activated carbon cloth. The resistant part of the circuit corresponds to a charge transfer phenomenon of electrocatalytic reactions. The resistance on the real axis describing the semicircle diameter known as charge transfer resistance (*R*_ct_) describes the interaction between the electrolyte and the electrode surface. Finally, at medium and low frequencies, two slopes appear with different inclinations. The first one corresponds to an anomalous diffusional process presented in porous electrodes^64−66^ like activated carbon cloth, modeled as CPE3-*R*_d_ in the equivalent circuit, where *R*_d_ is the diffusional resistance and a constant phase element (CPE3). The second slope corresponds to a double layer equilibrium, which can be found in the equivalent circuit as a constant phase element (CPE2).

The fitted curves obtained from EIS measurements of all the electrodes are shown in Figure S8, and Table S2 lists the fitting parameters. Some differences are noticed in the resistances presented above, especially in the charge transfer resistance (semicircle diameter, *R*_ct_), as can be seen in the bar plot shown in the inset of Figure 4a. The highest *R*_ct_ was recorded for the ACC electrode with a value of 7.295 Ω, which is slightly reduced when cerium oxide was loaded on the surface to 6.963 Ω and when the ACC was previously fluorinated (6.096 Ω). *R*_ct_ was reduced by 16% upon doping ACC with fluorine due to an enhancement in the electrode surface conductivity. However, a higher reduction was found for the CeO_2_/ACC-Pt electrode reaching a value of 5.222 Ω, 28% lower than the *R*_ct_ of ACC. According to this, an improvement in the interaction between the electrolyte and the electrode surface followed the order of CeO_2_/ACC-Pt > CeO_2_/ACC-F > CeO_2_/ACC > ACC. Hence, it is expected to observe a similar trend in electrocatalytic performance.

Linear sweep voltammetry curves are shown in Figure 4b, where the electrolyte pH was 11 at the beginning. Still, during the test, it was reduced to values around 6–7 due to the chlorine hydrolysis process and the corresponding hypochlorite formation (reactions 4 and 5). However, on the cathode surface, the pH is still alkaline due to the hydrogen evolution reaction’s production of OH- groups. A plateau area corresponding to a current-limiting region is commonly associated with the mass transport during the hypochlorite reduction (reaction 8). A constant potential of −0.6 V was chosen to study the performance of all the electrodes for hypochlorite reduction, as summarized in Table 3. A slight improvement was found when CeO_2_ was loaded on the ACC surface, but the plateau disappeared when ACC was doped with fluorine. Several explanations have been given when chromate compounds (Cr(VI)) are added to the electrolyte to avoid hypochlorite reduction. The principal argument is the formation of a chromium hydroxide (Cr(OH)_3_)/chromium oxide (Cr_2_O_3_) layer on the electrode surface that is considered to hinder the hypochlorite reduction acting as a diaphragm. A similar argument has been promulgated during the formation of lepidocrocite (γ-FeOOH) and some other alkaline hydroxides for different types of chlorate productions.^67−69^ Cerium oxide can quickly form a layer composed of cerium oxide and cerium hydroxide when immersed in an aqueous media that is favorable in an alkaline medium due to the presence of Ce^4+^ and Ce^3+^.^70^ In the presence of this layer, the electrode selectivity toward hypochlorite reduction decreases in favor of hydrogen evolution^71^ due to hydroxylation of the metal site blocking the active site for hypochlorite reduction.^18^

**Table 3:** Current Density Values Taken at −0.6 V (V vs Ag/AgCl) for Activated Carbon (ACC), Cerium Oxide Deposited on ACC (CeO_2_/ACC), CeO_2_ Deposited on Fluorinated ACC (CeO_2_/ACC-F), CeO_2_ on ACC with Platinum Nanoparticles Dispersed on It (CeO_2_/ACC-Pt) and Fluorinated ACC (ACC-F) Electrodes^a^

electrode	current density at −0.6 V (mA/cm^2^)
ACC	–16.617
CeO_2_/ACC	–13.260
CeO_2_/ACC-F	–8.785
CeO_2_/ACC-Pt	–14.318
ACC-F	–9.648

a The electrolyte used during the measurement corresponds to 2M NaCl + 80 mM NaOCl, potentials measured with respect to saturated Ag/AgCl reference electrode.

Figure S9 and Table 3 show that only the incorporation of fluorine into the ACC matrix produced a remarkable reduction in the hypochlorite reduction reaction. From the OCP values, it was found that the incorporation of fluorine atoms into ACC leads to the addition of negative charges on the electrode surface, agreeing with this observation since hypochlorite anions (OCl^–^) will be reduced (eq 8) on a negatively charged surfaces. This adverse potential gradient could affect the transportation rate and diffusion of OCl^–^^72^ hindering its reduction and decreasing the current density, corroborating well with the LSV results. Similar results have been found for fluorinated activated carbon in capacitive deionization applications (CDI), where the partial negative charge introduced due to fluorine groups enhances the cation adsorption and repelling anion adsorption on the cathode surface.^73,74^ The adverse potential becomes significant in materials with high surface area,^11^ like activated carbon.

**Figure 5:**
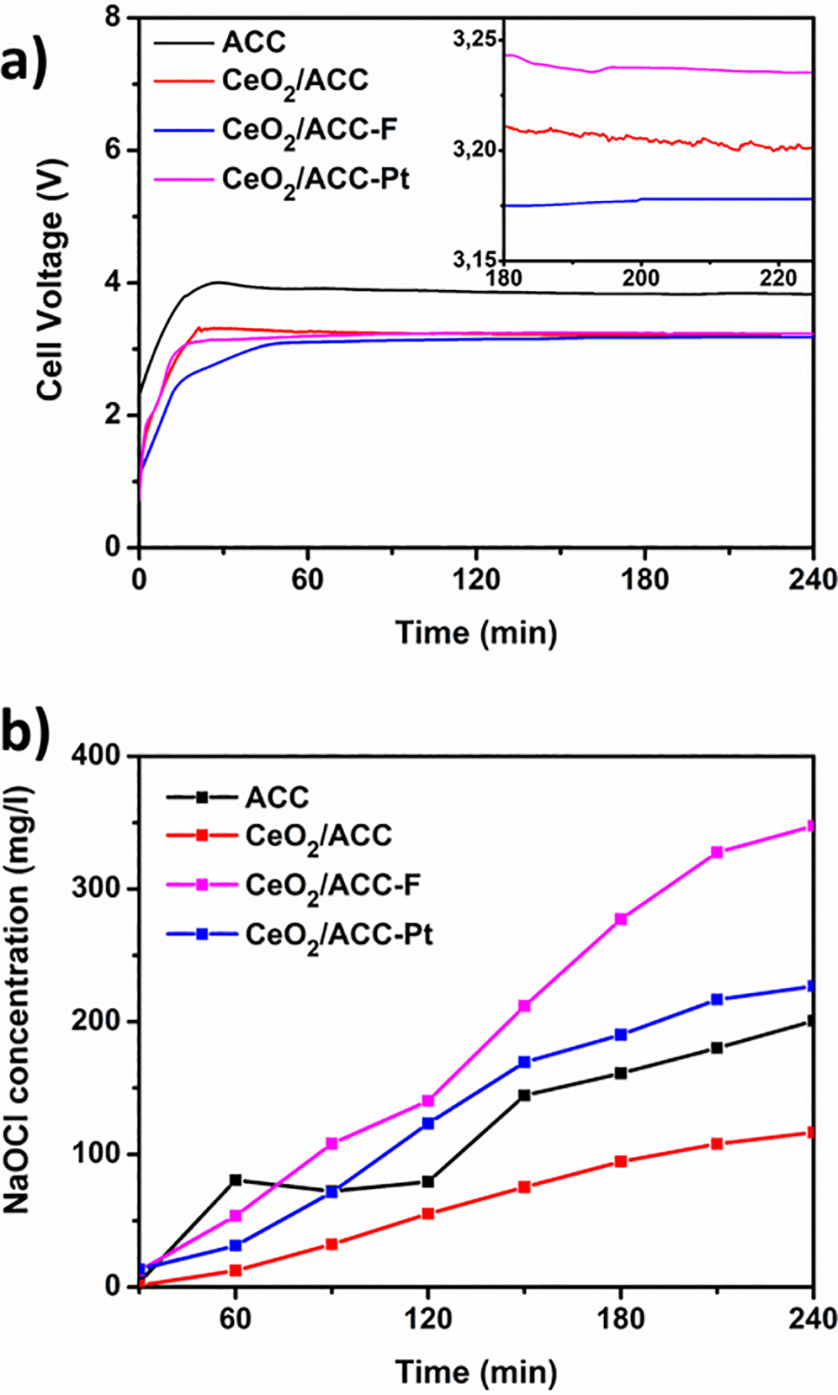
(a) Cell voltage recorded during the sodium hypochlorite production and (b) concentration of free chlorine produced during 240 min of performance for activated carbon cloth (ACC), cerium oxide growth on ACC (CeO_2_/ACC), CeO_2_ growth on fluorinated ACC (CeO_2_/ACC-F) and CeO_2_ growth on platinum nanoparticles deposited on ACC (CeO_2_/ACC-Pt). The starting solution corresponded to 30 g/L NaCl. The current density applied was 10 mA/cm^2^.

Sodium hypochlorite production was carried out in an undivided cell with two electrodes connected in parallel, separated by approximately 2 mm, where the electrolyte passed through both the electrodes. The current density used during the production of hypochlorite was 10 mA/cm^2^, and every test was carried out for 240 min. To compare the performance of the electrodes, the anode used in all the experiments was ACC without any modification. The cell voltage, as shown in Figure 5a, is unstable over a period in the beginning, and after around 40 min, it reaches an equilibrium value that remained constant during the rest of the test. Inset in Figure 5a shows the cell voltage in equilibrium, and it can be observed that all the electrodes present a stable value around 3.2 V except for symmetric ACC electrodes where a much higher constant work voltage of 4 V was recorded. Lower values of voltage can lead to better stability of the electrodes and higher durability over time. Figure 5b shows the hypochlorite production monitored every 30 min, where it is noticeable that the presence of CeO_2_ on ACC is lower than what is produced with ACC electrodes during the process even though the concentration of hypochlorite constantly grows. This can be explained by the double layer capacitance calculation discussed above. According to this, ECSA of CeO_2_/ACC is the lowest, which would thus interfere with the cathodic reaction and hypochlorite formation in the bulk solution.

It should be noted that in the first 30 min when ACC electrodes were used, higher production of hypochlorite (OCl^–^) occurs, followed by an abrupt fall and a slow rise thereafter; meanwhile, CeO_2_/ACC, CeO_2_/ACC-F, and CeO_2_/ACC-Pt electrodes show a continuous increase in production. The sodium hypochlorite concentration was higher for CeO_2_/ACC-F with 361 mg/L formed after 240 min, followed by CeO_2_/ACC-Pt with 226 mg/L, ACC 200 mg/L, and finally, CeO_2_/ACC electrodes producing only 116 mg/L. The lower hypochlorite production from CeO_2_/ACC electrodes can be related to its performance for hydrogen evolution. As shown in Figure 4b, the overpotential needed to reach at least 40 mA/cm^2^ is slightly higher than the ACC electrodes require. This is within our expectation since fluorine can hinder the hypochlorite reduction reaction more efficiently than just CeO_2_.

**Figure 6:**
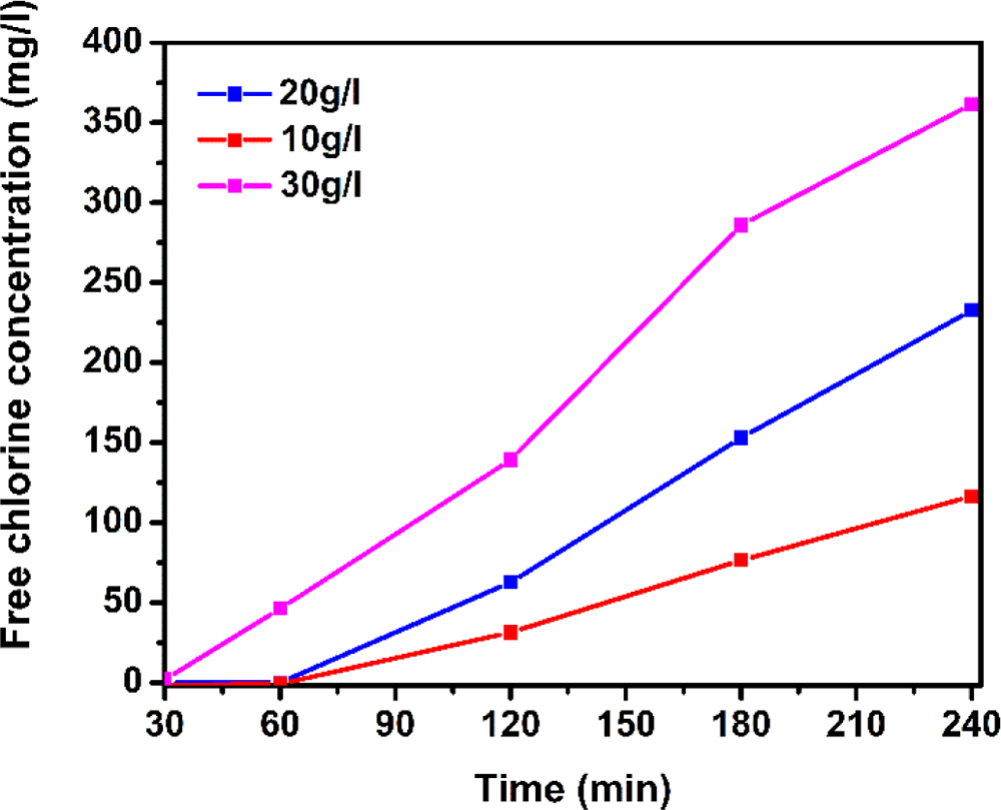
Free chlorine production at 10, 20, and 30 g/L NaCl concentration in the initial solution. The electrodes used corresponded to activated carbon cloth (ACC) as the anode and cerium oxide coating fluorinated ACC (CeO_2_/ACC-F) as the cathode. The current density applied was 10 mA/cm^2^.

Solutions with varying initial NaCl concentrations were tested using CeO_2_/ACC-F electrode as the cathode with pristine ACC anodes. Parameters were kept as in the previous experiments with a current density of 10 mA/cm^2^ and a pH of 9. From Figure 6, it can be noted that concentrations of 10 and 20 g/L of NaCl lead to a reduction in the hypochlorite production due to a lack of enough Cl^–^ ions for the chlorine evolution reaction. The final free chlorine concentrations were 116, 232, and 361 mg/L for 10, 20, and 30 g/L, respectively. Similar trends in free chlorine production were found in the literature when the main free chlorine species corresponded to hypochlorous acid (at acid pH).^75^

After the electrolysis test, the microstructure of the electrode was studied by scanning electron microscopy. From Figure S10, it is possible to determine that after using the electrodes, some changes were found in pristine ACC electrodes showing a nonuniform surface with marks like pores typical when activated carbon is treated in alkaline media.^76^ CeO_2_/ACC-Pt showed a significant detachment of the CeO_2_ layer. On the contrary, CeO_2_/ACC-F and CeO_2_/ACC showed almost no modification in their microstructure, showing better durability for long-term applications.

## 4 Conclusion

In this work, the hypochlorite reduction on activated carbon cloth (ACC) was studied using ACC electrodes modified by fluorination, platinum nanoparticles deposition, and a cerium oxide layer. When the surface of ACC was modified with cerium oxide, a decrease in the hypochlorite reduction was found, which could be attributed to arise from the formation of the Ce^4+^/Ce^3+^ layer. On the other hand, integration of fluorine into carbon structure leads to a partial negative surface charge incorporation, reflected in the open circuit potentials (OCP), showing values of 112 mV for CeO_2_/ACC-F, 90 mV for ACC-F, 87 mV for CeO_2_/ACC and 84 mV for CeO_2_/ACC-Pt, respectively. Similarly, the current density at −0.6 V reduced in the hypochlorite reduction region when ACC was modified with cerium oxide and fluorine with a maximum reduction for CeO_2_/ACC-F attributable to hypochlorite anion repulsion due to charged surface and hydroxylation of CeO_2_ layer. All of these lead to an improvement in cell voltage being reduced from 4 to 3.2 V and hypochlorite concentration produced during electrolysis, demonstrating that fluorination of activated carbon and coating the electrode with cerium oxide can be used to decrease the hypochlorite reduction rate and possibly can be an option to avoid the incorporation of Cr(VI) in chlorate process. The efficiency of the process can be further improved by modifying anode material and considering different degrees of heteroatom doping.

## Supporting Information Available

Additional experimental details and methods calibration (PDF)
