# Supplementary material for: Cerium Oxide on a Fluorinated Carbon-Based Electrode as a Promising Catalyst for Hypochlorite Production
Source: ACS Omega. 2022 Oct 10;7(42):37465–75. doi: 10.1021/acsomega.2c04248 (PMC9608405; doi:10.1021/acsomega.2c04248)
Supplement: Supplementary file 1 — ao2c04248_si_001.pdf [file ao2c04248_si_001.pdf]

Supporting information

Cerium oxide on Fluorinated carbon-based electrode as a promising catalyst for Hypochlorite production

*María Isabel Alvarado-Ávila, Esteban Toledo-Carrillo, Joydeep Dutta **

Functional NanoMaterials Group, Department of Applied Physics, School of Engineering Sciences, KTH Royal Institute of Technology, Hannes Alfvéns väg 12, 11419 Stockholm, Sweden

*Corresponding author.

e-mail address: joydeep@kth.se (J. Dutta)

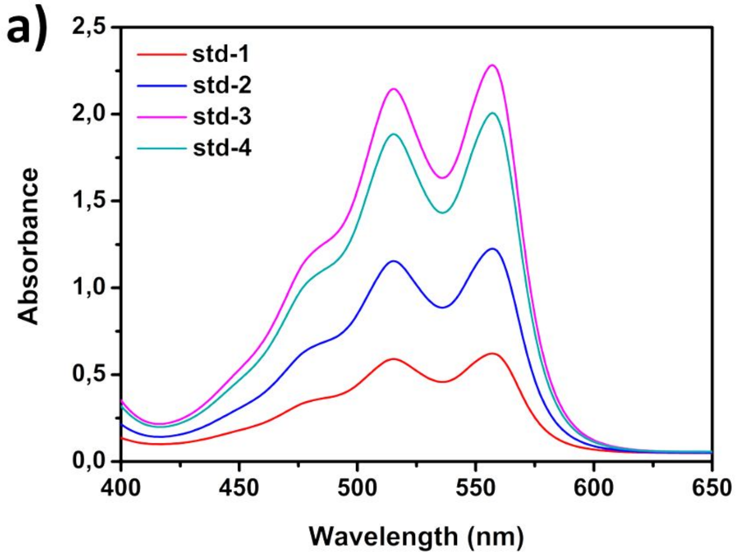

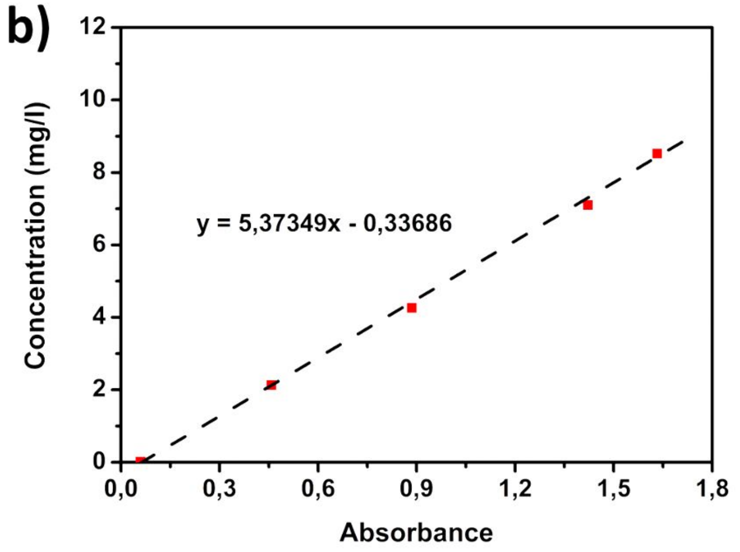

Figure S1 a) UV-Vis spectra of standard solution std-1 (2.13 mg/l), std-2 (4.26 mg/l), std-3 (7.1 mg/l) and std-4 (8.52 mg/l). b) calibration curve.

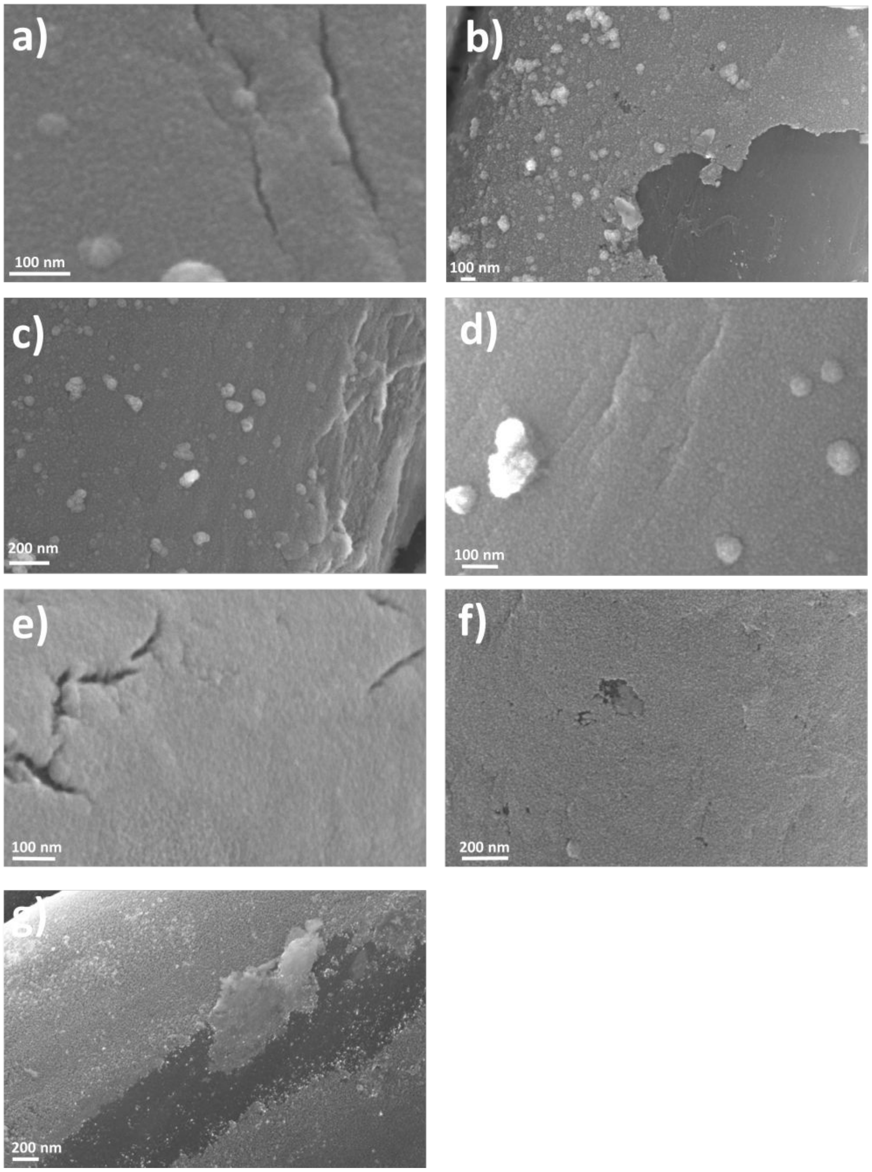

Figure S2 Cerium oxide layer deposition during reduction by 3M ammonia immersion at 50°C collected for different times. a) 15 min, b) 1 h, c) 3h, d) 6h, e) 12 h, f) 24 h, and g) after hydrothermal treatment.

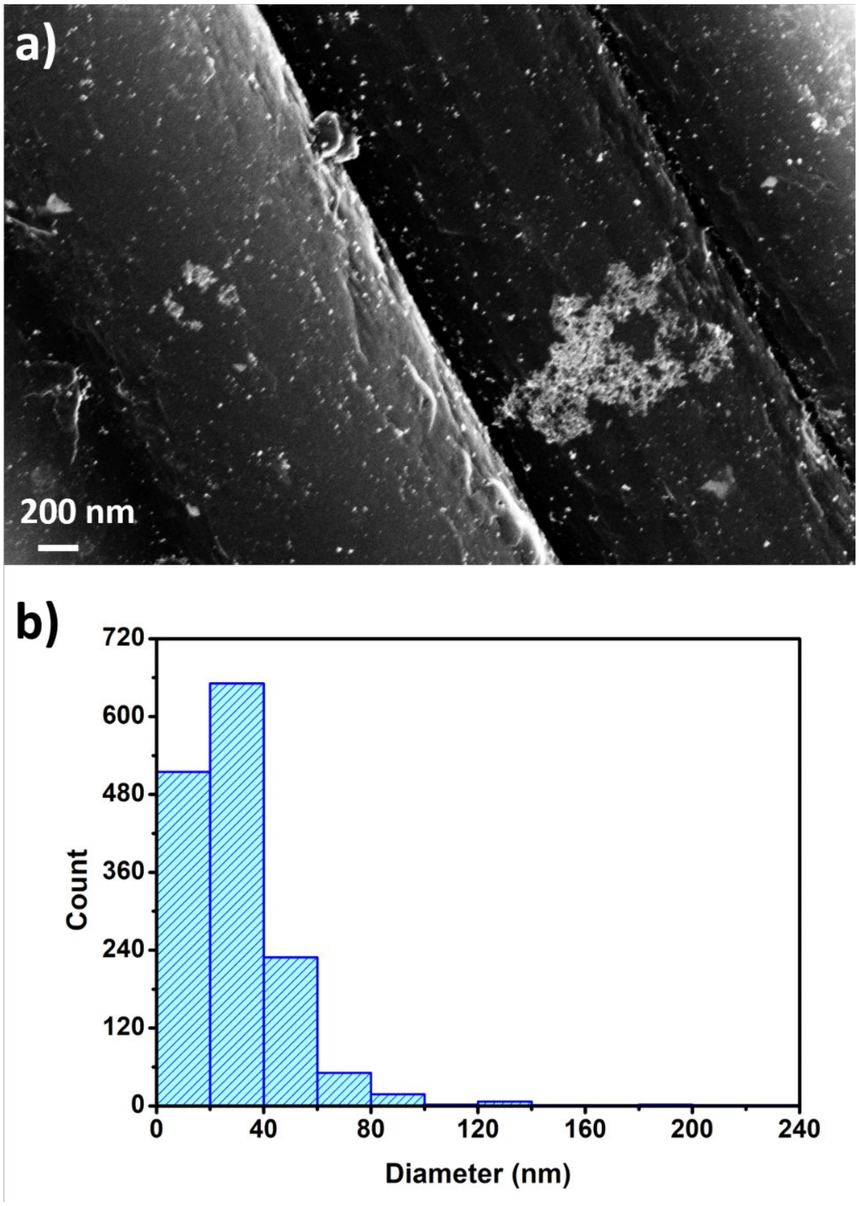

Figure S3 a) Agglomeration of platinum nanoparticles deposited on activated carbon cloth b) Distribution of particle sizes.

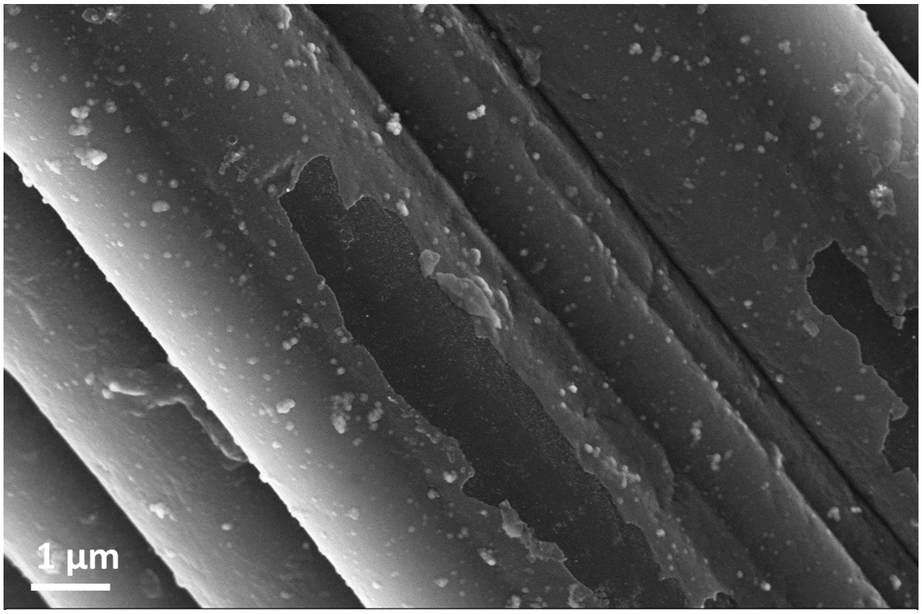

Figure S4 Cerium oxide layer on platinum nanoparticles deposited on activated carbon cloth.

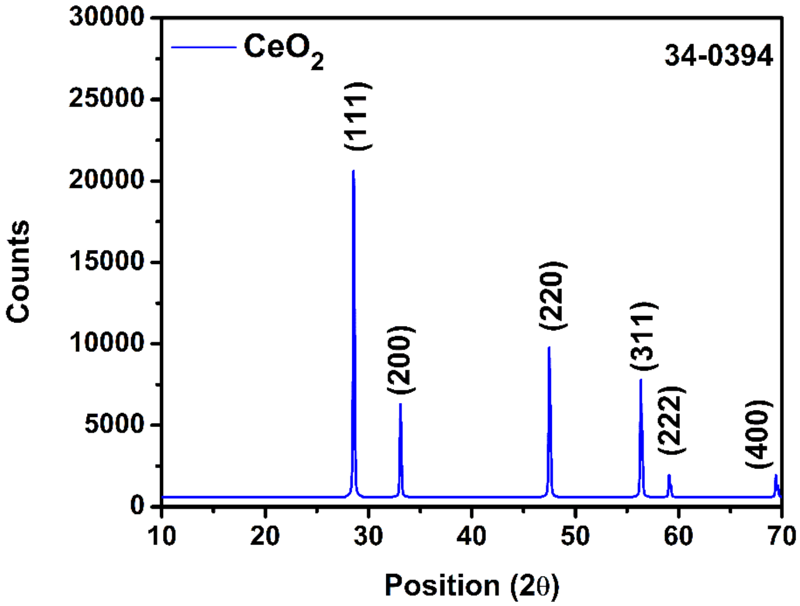

Figure S5. X-ray diffraction pattern JCPDS N° 34-0394 from cerium oxide

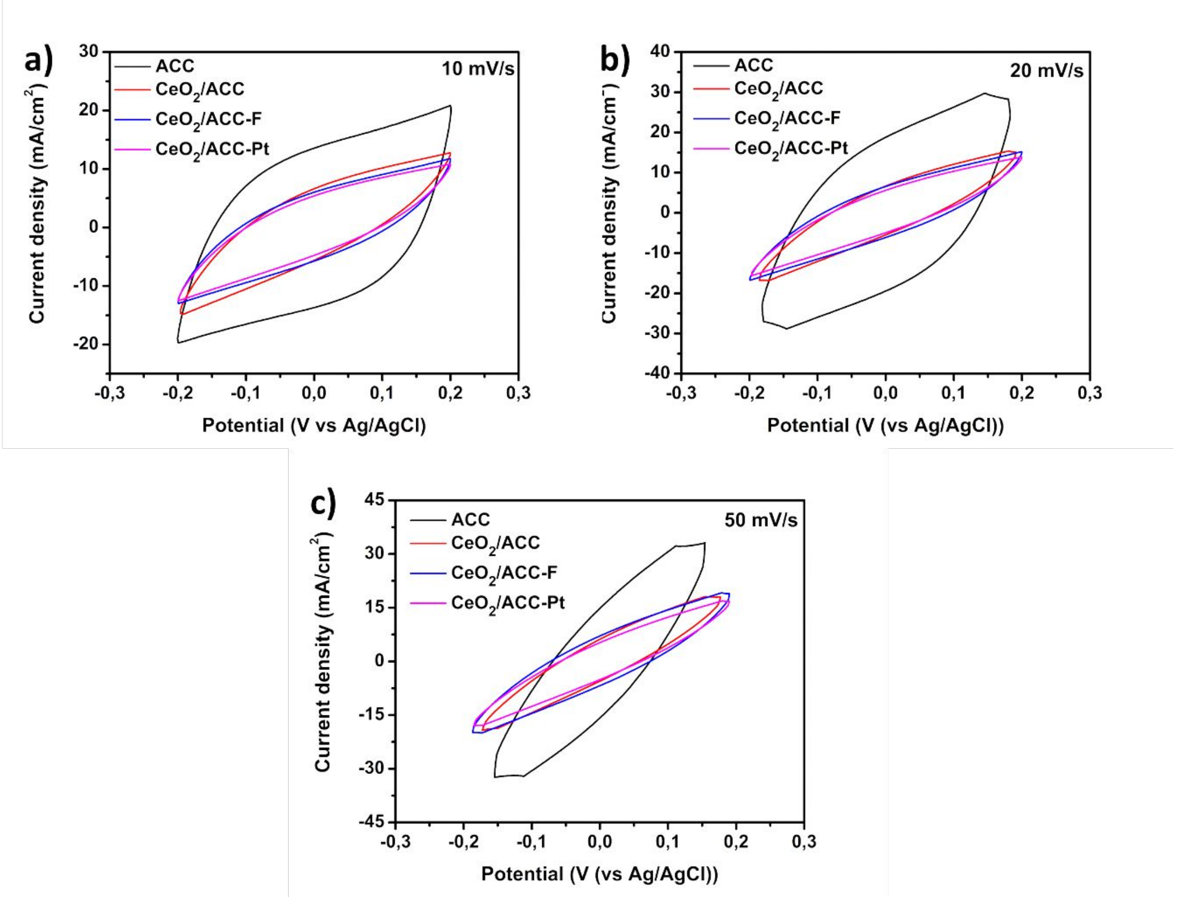

Figure S6 Cyclic voltammetry curves at different scan rates a) 10 mV/s, b) 20 mV/s, and c) 50 mV/s. For activated carbon cloth (ACC), Cerium oxide cover ACC (CeO_2/ACC), Cerium oxide covers fluorinated ACC (CeO_2/ACC-F), and cerium oxide on platinum nanoparticles deposited on ACC (CeO_2/ACC-Pt).

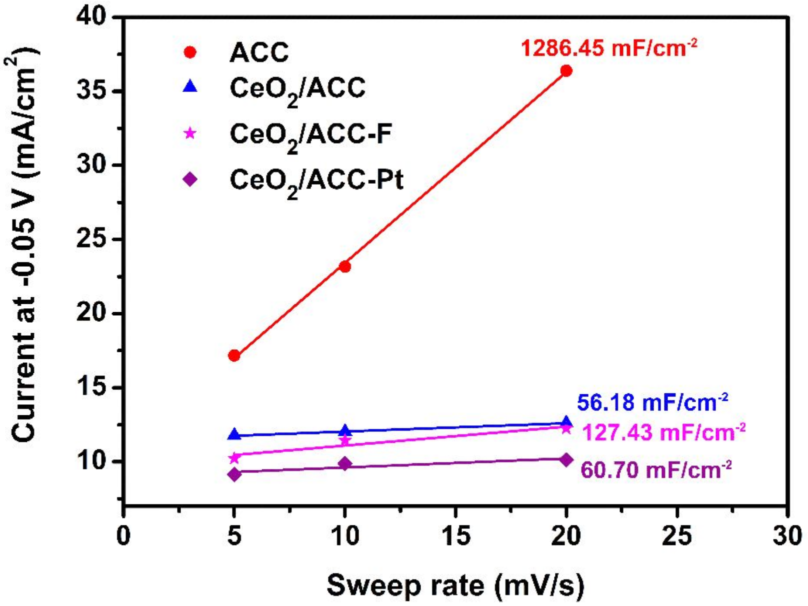

Figure S7 Current density at -0.05V measured from cyclic voltammetry vs. the scan rate. The straight line corresponds to linear fitting where the slope (mF/cm^2) represents the double layer capacitance (C_{DL}).

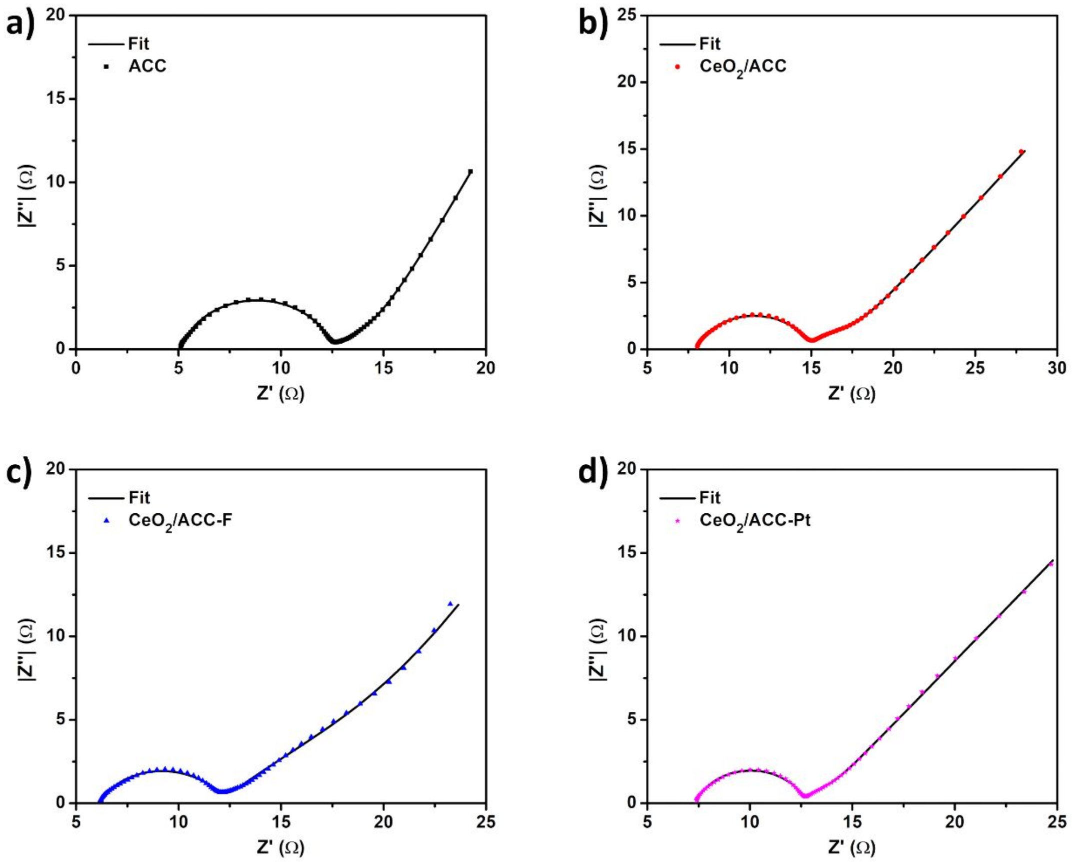

Figure S8 Electrochemical impedance spectroscopy (EIS) fitted curves using the equivalent circuit (R_s-(CPE1/(R_{ct}-(CPE3/R_d)))-CPE2) for the different electrodes a) Activated carbon cloth (ACC) b) Cerium oxide on ACC (CeO_2/ACC), c) Cerium oxide on fluorinated ACC (CeO_2/ACC-F) and d) cerium oxide on platinum nanoparticles deposited on ACC (CeO_2/ACC-Pt). Z' and Z'' are the real and imaginary impedance, respectively.

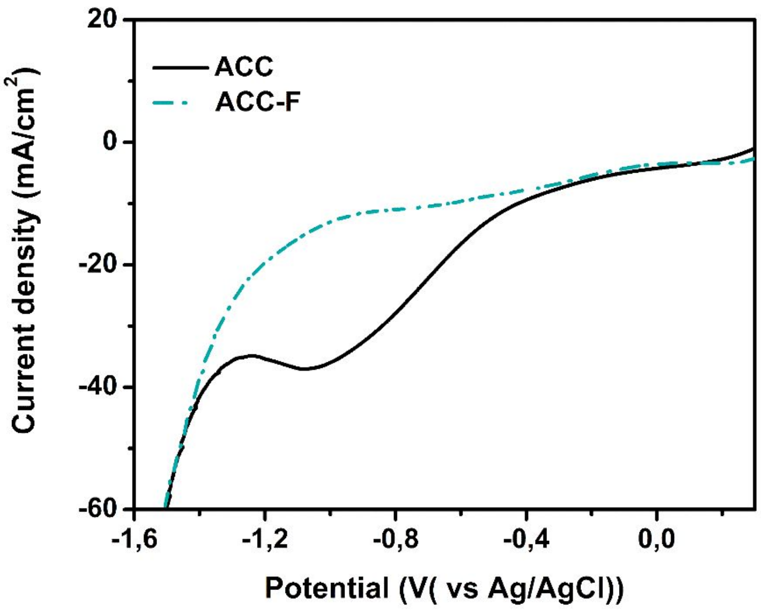

Figure S9 Linear sweep voltammetry (LSV) corresponding to activated carbon cloth (ACC) and fluorinated activated carbon cloth (ACC-F). Electrolyte 2M NaCl + 80 mM NaOCl.

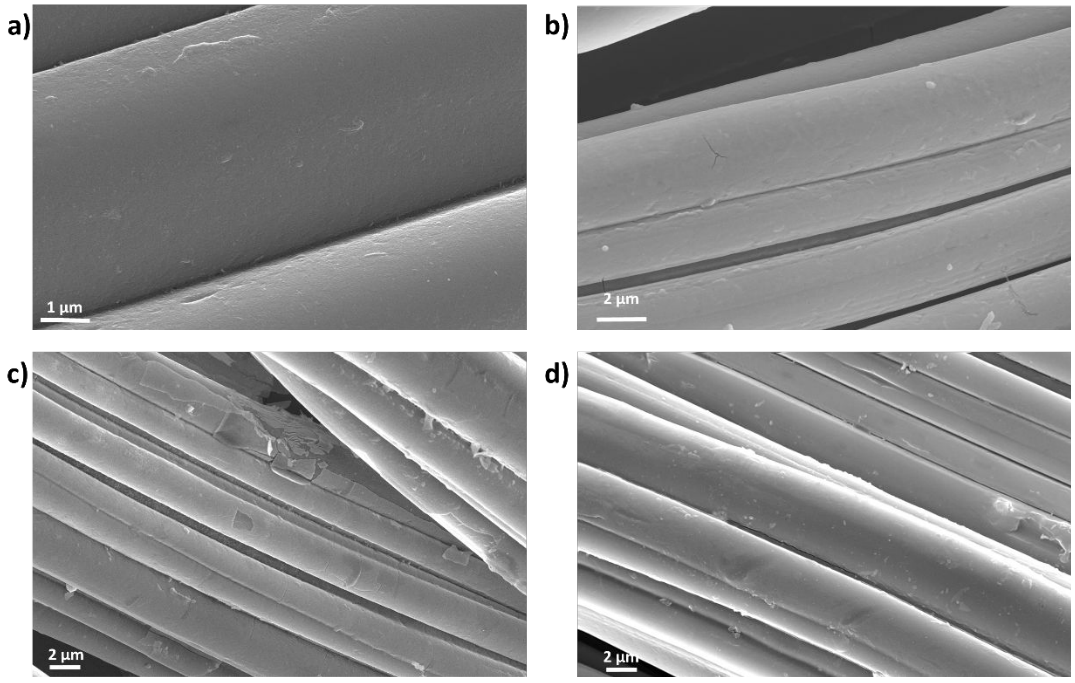

Figure S10 SEM micrograph of electrodes after hypochlorite production at 10mA/cm^2 for 4 hours. a) Activated carbon (ACC), b) cerium oxide on ACC (CeO_2/ACC), c) CeO_2 covered platinum nanoparticles on ACC (CeO_2/ACC-Pt) and d) CeO_2 covered fluorinated ACC (CeO_2/ACC-F)

Table S1 Fourier transform infrared (FTIR) band list and the corresponding group associated with each band for activated carbon cloth (ACC); cerium oxide coated ACC (CeO_2/ACC); cerium oxide coated fluorinated ACC (CeO_2/ACC-F), and cerium oxide coated ACC with platinum particles (CeO_2/ACC-Pt) electrodes.

Band (cm^{-1})	Electrode	Group	Reference
**3500-3000**	ACC; CeO_2/ACC; CeO_2/ACC-F; CeO_2/ACC-Pt	OH stretching vibration	
**1517 and 1530**	ACC; CeO_2/ACC; CeO_2/ACC-F; CeO_2/ACC-Pt	N-O stretching vibration (absorbed on electrodes' surfaces during nitric acid treatment)	1
**1400 to 1000**	ACC; CeO_2/ACC; CeO_2/ACC-F; CeO_2/ACC-Pt	C-O stretching vibration (ester, ethers, and carboxylic acid)	2
**1095**	CeO_2/ACC-F	C-F semi-covalent bond	3
**815 - 725**	CeO_2/ACC; CeO_2/ACC-F; CeO_2/ACC-Pt	Ce-O stretching vibration	4, 5

Table S2 Fitted parameters for Nyquist plot obtained using the EIS spectrum analysis software. Where Q^0 and n are the pre-factor and the exponent of the CPE, respectively. When n is closer to 1, the performance of the electrode is more capacitive meanwhile when n is closer to 0, the material presents a resistant behavior

**Parameters**	**Rs**	**Rct (Ω)**	**Rd (Ω)**	**CPE1**		**CPE2**		**CPE3**	
**Electrode**	**(Ω)**			**Q_1^0(Ω^{-1} s^n)**	**n1**	**Q_2^0 (Ω^{-1} s^n)**	**n2**	**Q_3^0 (Ω^{-1} s^n)**	**n3**
**ACC**	5.147	7.295	2.337	0.0000557	0.860	0.654	0.728	0.271	0.503
**CeO_2/ACC**	7.980	6.963	2.170	0.000119	0.790	0.283	0.596	0.103	0.670
**CeO_2/ACC-F**	6.113	6.096	7.662	0.000342	0.713	0.555	0.685	0.224	0.541
**CeO_2/ACC-Pt**	7.368	5.222	1.604	0.000102	0.813	0.292	0.599	0.340	0.488

References

1. Rodriguez, R.; Contrino, D.; Mazyck, D., Role of Activated Carbon Precursor for Mercury Oxidation and Removal: Oxidized Surface and Carbene Site Interaction. *Processes* **2021,** *9* (7), 1190.
2. El-Shafey, E. I.; Ali, S. N. F.; Al-Busafi, S.; Al-Lawati, H. A. J., Preparation and characterization of surface functionalized activated carbons from date palm leaflets and application for methylene blue removal. *Journal of Environmental Chemical Engineering* **2016,** *4* (3), 2713-2724.
3. Khabashesku, V. N.; Billups, W. E.; Margrave, J. L., Fluorination of single-wall carbon nanotubes and subsequent derivatization reactions. *Acc. Chem. Res.* **2002,** *35* (12), 1087-1095.
4. Kumar, E.; Selvarajan, P.; Balasubramanian, K., Preparation and studies of cerium dioxide (CeO2) nanoparticles by microwave-assisted solution method. *Recent Res. Sci. Technol.* **2010,** *2* (4).
5. Pop, O. L.; Diaconeasa, Z.; Mesaroş, A.; Vodnar, D. C.; Cuibus, L.; Ciontea, L.; Socaciu, C., FT-IR studies of cerium oxide nanoparticles and natural zeolite materials. *Bull. UASVM Food Sci. Technol.* **2015,** *72* (1), 50-55.
